# 

**Published:** 1839-09

**Authors:** 


VOL I.—NO. III.

EDITED BY

CHAPIN A. HARRIS, M. D.
BALTIMORE.

ELEAZAR PARMLY,
NEW-YORK.

PUBLISHING COMMITTEE,

E. PARMLY, | E. BAKER,
S. BROWN.

NEW YORK,

GEORGE ADLARD, 168 BROADWAY,

BALTIMORE,

ARMSTRONG & BERRY.

PRICE---THREE DOLLARS PER ANNUM.

KELLEY & FRAETAS, PRINTERS,

